# BST2 Mediates Osteoblast Differentiation *via* the BMP2 Signaling Pathway in Human Alveolar-Derived Bone Marrow Stromal Cells

**DOI:** 10.1371/journal.pone.0158481

**Published:** 2016-06-30

**Authors:** Su-Hyang Yoo, Jae Goo Kim, Beom-Su Kim, Jun Lee, Sung-Hee Pi, Hyun-Dae Lim, Hong-In Shin, Eui-Sic Cho, Hyung-Keun You

**Affiliations:** 1 Department of Periodontology, School of Dentistry, Wonkwang University, Iksan, Korea; 2 Faculty of Biological Science and Institute for Biodiversity Research, College of Natural Sciences, Chonbuk National University, Jeonju, Korea; 3 Wonkwang Bone Regeneration Research Institute, Wonkwang University, Daejeon, Korea; 4 Bone Cell Biotech Inc., Daejeon, Korea; 5 Department of Oral and Maxillofacial Surgery, Daejeon Dental Hospital, Wonkwang University, Daejeon, Korea; 6 Department of Oral Medicine, School of Dentistry, Wonkwang University, Iksan, Korea; 7 IHBR, Department of Oral Pathology, School of Dentistry, Kyungpook National University, Daegu, South Korea; 8 Cluster for Craniofacial Development and Regeneration Research, Institute of Oral Biosciences, Chonbuk National University School of Dentistry, Jeonju, Korea; Kyungpook National University School of Medicine, REPUBLIC OF KOREA

## Abstract

The molecular mechanisms controlling the differentiation of bone marrow stromal stem cells into osteoblasts remain largely unknown. In this study, we investigated whether bone marrow stromal antigen 2 (*BST2*) influences differentiation toward the osteoblasts lineage. *BST2* mRNA expression in human alveolar-derived bone marrow stromal cells (hAD-BMSCs) increased during differentiation into osteoblasts. hAD-BMSCs differentiation into osteoblasts and the mRNA expression of the bone-specific markers alkaline phosphatase, collagen type α 1, bone sialoprotein, osteocalcin, and osterix were reduced by *BST2* knockdown using siRNA. Furthermore, *BST2* knockdown in hAD-BMSCs resulted in decreased *RUNX2* mRNA and protein expression. We hypothesized that *BST2* is involved in differentiation of into osteoblasts *via* the *BMP2* signaling pathway. Accordingly, we evaluated the mRNA expression levels of *BMP2*, BMP receptors (*BMPR1* and *2*), and the downstream signaling molecules SMAD1, SMAD4, and p-SMAD1/5/8 in *BST2* knockdown cells. *BMP2* expression following the induction of differentiation was significantly lower in *BST2* knockdown cells than in cells treated with a non-targeting control siRNA. Similar results were found for the knockdown of the *BMP2* receptor- *BMPR1A*. We also identified significantly lower expression of SMAD1, SMAD4, and p-SMAD1/5/8 in the *BST2* knockdown cells than control cells. Our data provide the first evidence that *BST2* is involved in the osteogenic differentiation of bone marrow stromal cells *via* the regulation of the *BMP2* signaling pathway.

## Introduction

Osteoblasts differentiate from bone marrow stromal cells (BMSCs), also known as mesenchymal stem cells, which have the capacity to become adipocytes or fibroblasts [1]. In recent years, human alveolar-derived BMSCs (hAD-BMSCs) have been successfully isolated and cultured [2]. These cells may be useful for periodontal bone regenerative medicine because marrow blood can be easily aspirated from alveolar bone during tooth extraction and dental implant surgery [3, 4].

The bone morphogenetic protein (BMP) 2 signaling pathway is an essential regulator of osteogenesis. BMP2 binds to its receptors and activates SMADs, which directly regulate target gene expression [5]. BMP2 activates BMP receptors (BMPRs) 1 and 2 to initiate signal transduction. Activated BMPR1 phosphorylates receptor-specific SMAD 1, 5, and 8, each of which form complexes with SMAD 4 [6, 7]. The target genes of BMP2 in osteoblasts encode various transcription factors, such as DLX3, DLX5, ATF4, runt-related transcription factor-2 (RUNX2), and osterix (OSX) [8]. In particular, *RUNX 2* is a key transcription factor for osteogenesis [9], and regulates the expression of several osteoblastic genes including collagen type 1α (*COL1α*), bone sialoprotein (*BSP*) and the skeletal-specific osteocalcin (*OCN*) gene. However, little is known about the specific signaling pathways that control gene expression during the differentiation of osteoblasts from BMSCs. In a previous study, we screened for genes that are differentially expressed between undifferentiated hAD-BMSCs and fully differentiated osteoblasts [10]. We identified several differentially expressed genes, including bone marrow stromal antigen 2 (*BST2*).

BST2 expression affects cancer cell behavior [11], retains budding viruses to the cell plasma membrane, and inhibits virus replication [12, 13]. *BST2* expression was initially identified in human differentiated B cells, plasma cell lines, and myeloma cells [14, 15]. Recently, *BST2* was designated *CD317* and found to be widely expressed in all stages of B cell differentiation as well as in T cells, monocytes, CD34^+^ progenitorcells, and non-hematopoietic cells in humans [16]. Furthermore, BST-2 expression by BMSCs could promote the growth of murine pre-B cells [17]. However, the role of *BST2* in the differentiation of osteoblasts from BMSCs is unclear. The purpose of this study was to evaluate the functions and signal transduction pathways associated with *BST2* during the differentiation of osteoblasts from hAD-BMSCs.

## Materials and Methods

### Culture of hAD-BMSCs and the induction of osteoblast differentiation

To obtain hAD-BMSCs, alveolar bone marrow aspirates (0.5–1.0 mL) were collected from osteotomy sites during implant surgery using an 18-gauge needle syringe. The patients were 50–60 years of age (n = 4). All MSC donors provided written informed consent. Patient recruitment and the study protocols were approved by the Institutional Review Board at the Wonkwang University Dental Hospital (WKDIRB201403-02). hAD-BMSCs were isolated and expanded as described previously [2]. To induce osteoblast differentiation, cells (nearly 90% confluent) were treated with osteoblast-induction stimulants (OS) containing 10 mM β-glycerophosphate, 50 μg/mL ascorbic acid, and 100 nM dexamethasone (Sigma-Aldrich, St. Louis, MO, USA). The medium and OS were refreshed every 2 days after initial plating.

### Knockdown of *BST2* using siRNA

Two siRNAs specifically targeting *BST2* and a negative control siRNA were designed and synthesized by Bioneer (Daejeon, Korea; catalogue numbers 1013484 and 1013488). Cells were transfected with siRNA using Lipofectamine^®^ 2000 (Invitrogen, Carlsbad, CA, USA) following the manufacturer’s protocol. To confirm the efficiency of siRNA-mediated knockdown, *BST2* mRNA and protein levels were evaluated by quantitative real-time PCR (qRT-PCR) and immunoblotting, respectively.

### Semi quantitative PCR and qRT–PCR assays

Total RNA was extracted from cultured cells using TRIzol reagent (Invitrogen) according to the manufacturer’s protocol and quantified with a Nano-drop 2000 (Thermo Fisher Scientific, Waltham, MA, USA). First-strand cDNA was synthesized with the PrimeScript^™^ RT Reagent Kit (Takara Bio, Otsu, Japan). Semi-quantitative PCR was performed with HiPi^™^ 5× PCR Premix (Elpis Biotech, Daejeon, Korea) with *GAPDH* as the control gene. After amplification, PCR products were separated by electrophoresis on a 1% (w/v) agarose gel dyed with 0.5 μL/mL ethidium bromide, and gel images were obtained using an imaging system (RED^™^, Alpha Innotech, San Leandro, CA, USA) and saved in the JPG file format. Then, the signal intensity of the captured images was quantified using ImageJ (NIH, Bethesda, MD, USA). The relative densities were estimated as the ratios of the signal intensities of the bands corresponding to *BST2*, *RUNX2*, *BMP2*, *BMPR1A*, *BMPR1B*, and *BMPR2* to that of the band corresponding to *GAPDH*. qRT-PCR was performed with HiPi^™^ Real-time PCR 2× Master Mix (Elpis Biotech) with *GAPDH* as an internal control. To determine the expression levels of *COL1α*, *BSP*, *OCN*, *OSX*, *ALP*, *RUNX2*, and *BST2*, the cDNA samples were analyzed by qRT-PCR using an ABI PRISM^®^ 7300 unit (Applied Biosystems, Foster City, CA, USA). Primers for the genes are listed in Table 1. Qualitatively similar results were obtained using semi-quantitative PCR and qRT-PCR assays, confirming that our analyses were appropriate.

**Table 1:** Primers Used for Semi-Quantitative PCR and Real-Time PCR (qRT-PCR).

Gene	Sequence (5′-3′)	
	Sense primer	Antisense primer
***ALP***	ACGTGGCTAAGAATGTCATC	CTGCTAGGCGATGTCCTTA
***ColⅠ-α***	GATGGATTCCAGTTCGAGTATG	GTTTGGGTTGCTTGTCTGTTTG
***Bsp***	TCAGCATTTTGGGAATGGCC	GAGGTTGTTGTCTTCGAGGT
***OCN***	ATGAGAGCCCTCACACTCCTC	CGTAGAAGCGCCGATAGGC
***Runx2***	CGAATGGCAGCACGCTATTAA	GTCGCCAAACAGATTCATCCA
***OSX***	TAATGGGCTCCTTTCACCTG	CACTGGGCAGACAGTCAGAA
***BMP2***	AGTTGCGGCTGCTCAGCATGTT	CCGGGTTGTTTTCCCCACT
***BMPR1 A***	TTTATGGCACCCAAGGAAAG	TGGTATTCAAGGGCACATCA
***BMPR1 B***	AAAGGTCGCTATGGGGAAGT	GCAGCAATGAAACCCAAAAT
***BMPR2***	CATCCGAACCCTCTCTTGAT	TGCATAAAGATCCATTGGGA
***GAPDH***	GTCAGTGGTGGACCTGACCT	AGGGGAGATTCAGTGTGGTG

### Alkaline phosphatase (ALP) activity assay and staining

ALP activity was determined at 5 days after treatment with OS using *p*-nitrophenylphosphate as a substrate, as described previously [10]. ALP activity was normalized to the total protein content as measured using a bicinchoninic acid protein assay (Pierce, Rockford, IL, USA). The data are presented as nmol/30 min/mg of protein. Experiments were repeated 4 times. To examine ALP activity by histochemistry, the cells were stained with 0.1 mg/mL naphthol AS-MX phosphate (Sigma-Aldrich) and 0.6 mg/mL Fast Red Violet LB Salt (Sigma-Aldrich) after 5 days of culture and then photographed with a digital camera (D80: Nikon, Tokyo, Japan).

### Calcium accumulation assay

Calcium accumulation was determined using alizarin red-sulfate (AR-S, Sigma-Aldrich). hAD-BMSCs were cultured in 24-well plates for 21 days with continuous OS treatment. Then, cells were fixed with ice-cold 70% (v/v) ethanol for 1 h at 4°C. The ethanol was then removed and 40mM AR-S staining solution was added to cells for 1 min at room temperature. The stained cells were photographed using a digital camera (D80, Nikon).

### Immunoblotting

For the western blot analysis, each protein extract (30 μg of total protein) was resolved by SDS-PAGE using 10% polyacrylamide gels and subsequently transferred to Immun-Blot^®^ PVDF membranes (Bio-Rad, Hercules, CA, USA). The primary antibodies and their dilution factors were as follows BST2, 1:1000 (Santa Cruz Biotechnology, Dallas, TX, USA); RUNX2, 1:1000 (Santa Cruz Biotechnology); SMAD1, 1:1000 (Abcam, Cambridge, UK); SMAD4, 1:500 (Abcam); SMAD5. 1:500 (Abcam); SMAD 8, 1:500 (Abcam); T-SMAD1/5/8, 1:500 (Santa Cruz Biotechnology); p-SMAD1/5/8, 1:500 (Santa Cruz Biotechnology), and β-actin, 1:1000 (Sigma-Aldrich). Secondary antibodies were used at a 1:5000 dilution. The detection of protein bands was facilitated by an Enhanced Chemiluminescence Kit (Elpis Biotech) and membranes exposures to X-ray film (Amersham, Buckinghamshire, UK).

### Immunohistochemistry of BST2

Human bone tissues were perfusion-fixed with 10% buffered formalin. Bone tissues sections (4 μm) were stained with hematoxylin–eosin. For immunohistochemical staining, sections were deparaffinized, rehydrated with phosphate-buffered saline and incubated for 30 min at 37°C with primary BST2, 1:200 (Santa Cruz Biotechnology) followed by staining using a streptavidin biotin labeling kit (DAKO Corp., Glostrup, Denmark). Section were analyzed using a IX71 microscope (OLYMPUS, Tokyo, Japan) connected to a DP72 camera (OLYMPUS) which allowed the projection of the entire field of interest onto a computer monitor.

### Statistical analysis

The experimental results are expressed as means ± standard error of the mean (SEM). One-way analysis of variance was used for multiple comparisons, followed by Dunnett’s tests. *P* values< 0.05 and < 0.01 were considered significant.

## Results

### *BST2* expression was inhibited by siRNA

**Fig 1:**
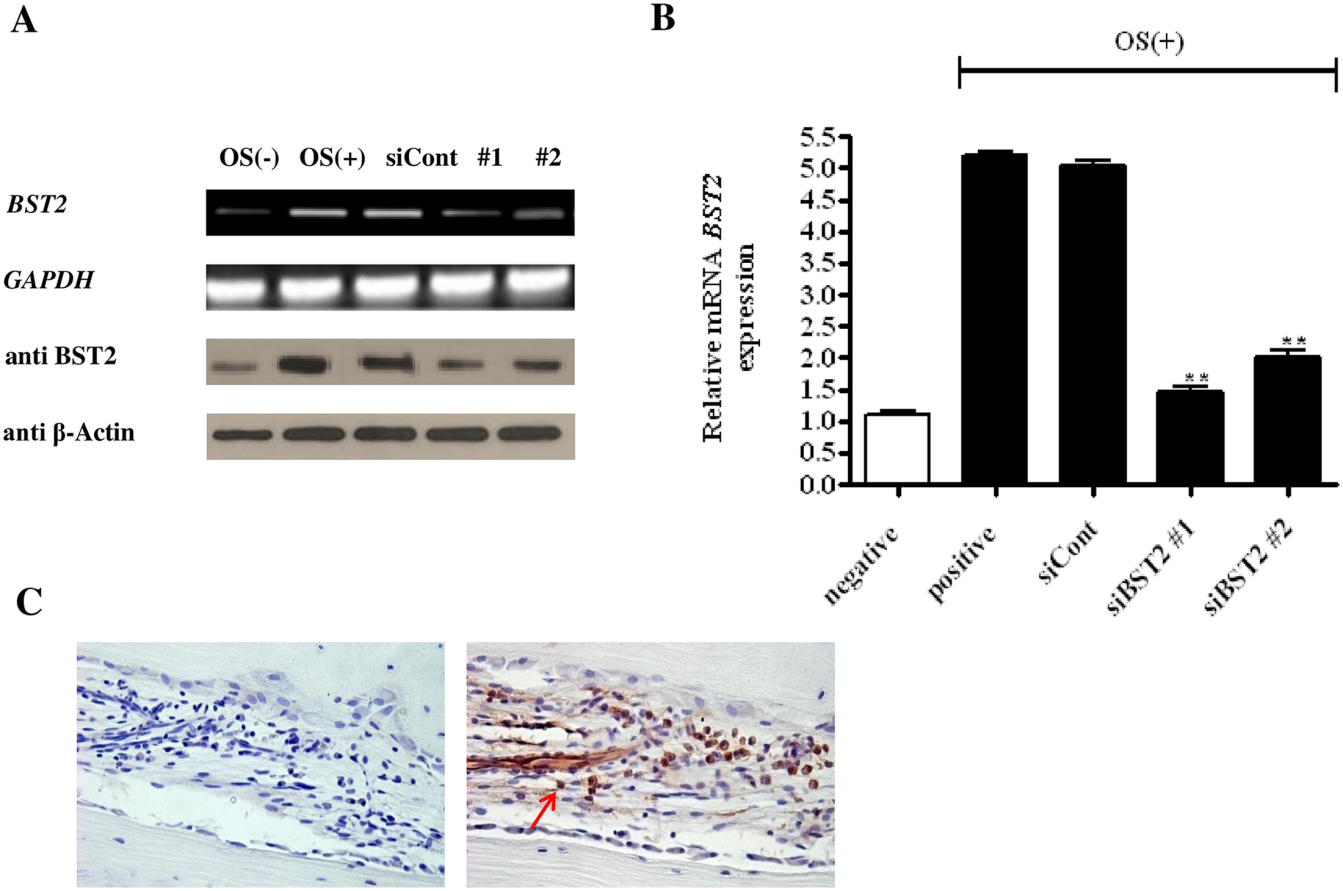
Effects of *BST2* knockdown by siRNA on *BST2* expression in hAD-BMSCs. RT-PCR and immunoblotting analyses (A) as well as qRT-PCR (B) demonstrated that the expression of *BST2* was lower in cells treated for 5 days with OS and si*BST2* #1 or #2 than in cells treated with OS and non-targeting siRNA (siCont). (C) Expression of BST2 in the human bone marrow. Representative immunostaining of BST2 in the human bone marrow was observed under light microscope (magnification ×400). Brown staining (marked with a red arrow) indicates the expression of BST2 in bone marrow. Values are expressed as means ± SEM (standardized mean difference) **, *P*<0.01 compared withthe expression level in cells treated with the control siRNA (siCont) (n = 4).

*BST2* protein and mRNA were expressed at basal levels in OS-untreated cells and increased after OS treatment. *BST2* was only minimally expressed in untreated cells, but its expression was significantly higher in OS-treated cells (Fig 1A). These results indicated that *BST2* expression was significantly increased during the differentiation of hAD-BMSCs into osteoblasts. To determine the influence of *BST2* knockdown on osteoblast differentiation, cells transfected with siRNA were cultured in the presence or absence of OS. *BST2* expression was continuously inhibited in cells treated with OS and either si*BST2* #1 or #2 compared with cells treated with OS and non-targeting siRNA (Fig 1B). qRT-PCR data showed that *BST2* mRNA expression was reduced in si*BST2* #1 and #2 treated cells by approximately 74% and 63%, respectively, compared with cells treated with the control siRNA (Fig 1B). We also were confirmed the expression of BST2 in human bone tissue that was surgically removed from patients (Fig 1C).

### *BST2* knockdown inhibited ALP activity and calcium accumulation in osteoblasts

**Fig 2:**
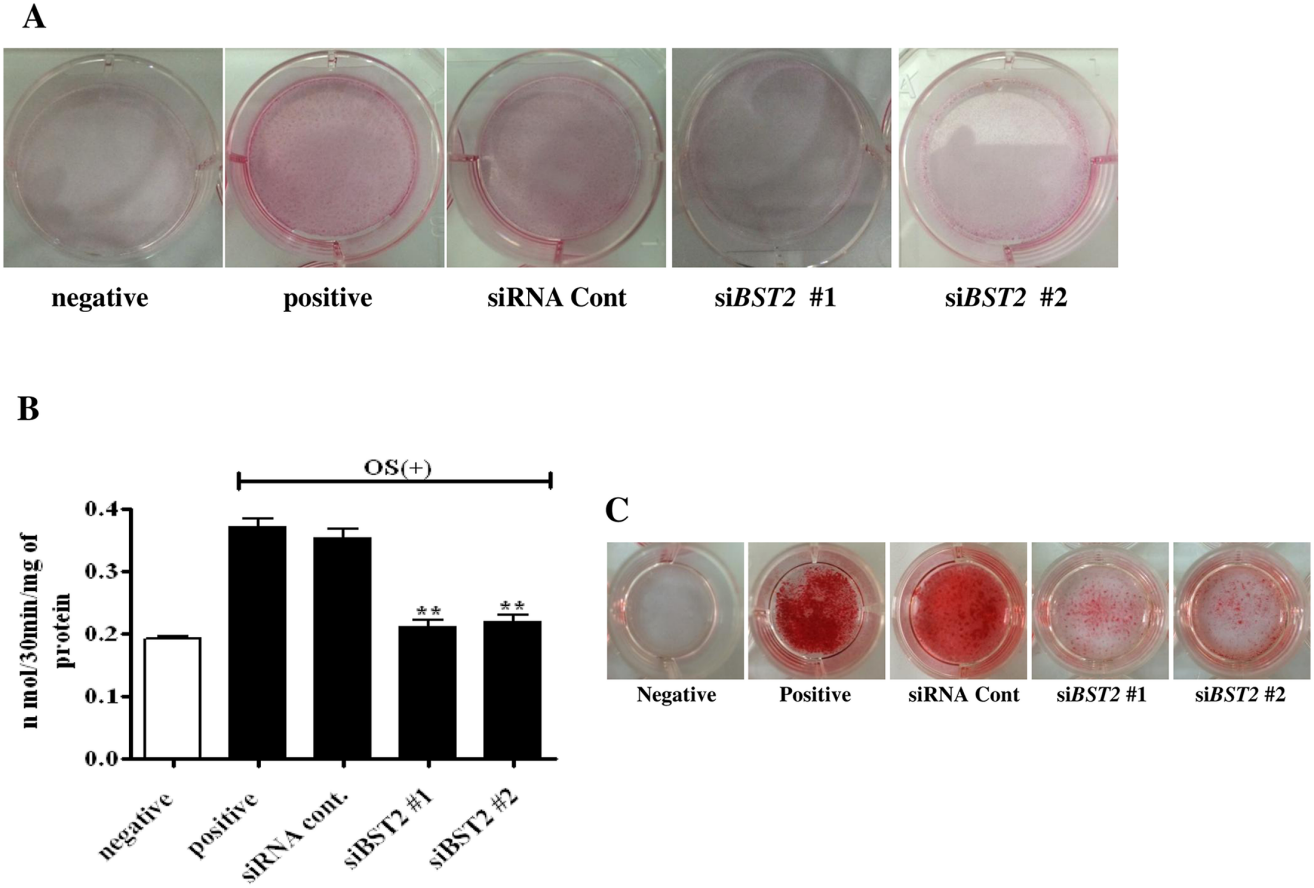
Effects of *BST2* knockdown on osteoblast differentiation in hAD-BMSCs. ALP staining (A) and ALP activity (n = 4) (B) were reduced after *BST2* knockdown. At 21 days of cultivation, *BST2* knockdown by siRNA resulted in a significant reduction in AR-S staining for calcium accumulation (C). Values are expressed as means ± SEM. **, *P*<0.01 compared with the control siRNA-treated group (siRNA Cont).

OS-treated control cells showed strong ALP staining, whereas cells treated with OS and either si*BST2* #1 or si*BST2* #2 showed weak ALP staining (Fig 2A). ALP activity was significantly higher in OS-treated controls cells than in OS-untreated controls cells; however, the knockdown of *BST2* resulted in significant decreases in ALP activity in cells treated with OS + si*BST2* #1 (0.16 ± 0.01μmol/30 min/mg of protein) and OS + si*BST2* #2 (0.15 ± 0.01μmol/30 min/mg of protein) compared with the activity levels of cells treated with OS and the control siRNA (by approximately 3.6-fold and 4.5-fold, respectively) (Fig 2B). In addition, AR-S staining showed large decreases in calcium accumulation in cells treated with OS and si*BST2* #1 or si*BST2* #2 compared with cells treated with OS and the control siRNA (Fig 2C).

### *BST-2* knockdown inhibited the expression of osteoblast markers

**Fig 3:**
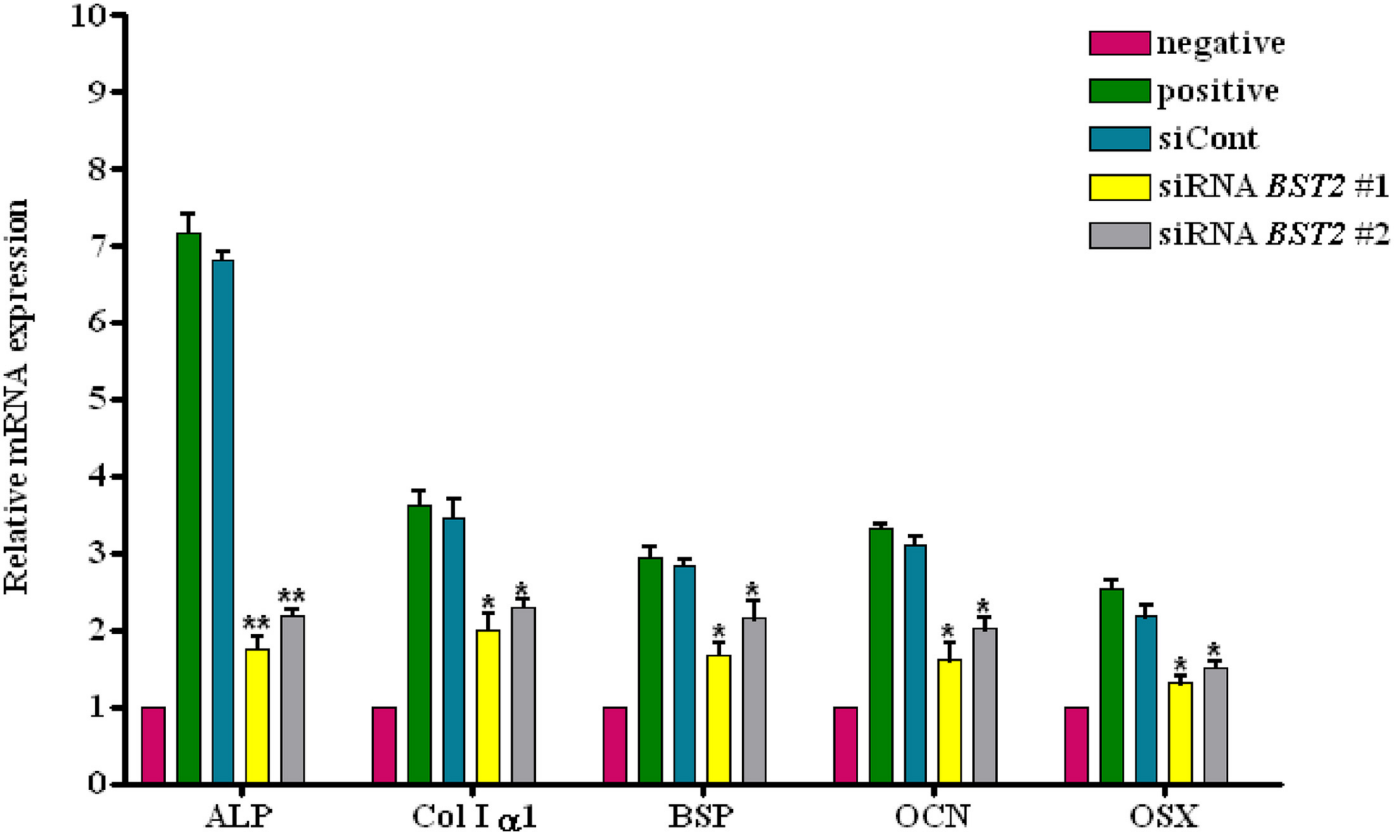
Expression of osteoblast differentiation markers following *BST2* knockdown. hAD-BMSCs transfected with siRNA were cultured for 5 days in the presence or absence of OS, and qRT-PCR was performed to measure the expression of *ALP*, *COL1α1*, *BSP*, *OCN*, and *OSX*, with *GAPDH* as the internal control. Relative expression levels were normalized to the expression of the untreated negative control sample. Compared with the untreated control, increased expression levels of *ALP*, *OSX*, *COL1α1*, *BSP*, and *OCN* were apparent in cells treated with OS and the control siRNA; however, the expression of these markers was decreased after *BST2* knockdown by siRNA. Values are expressed as means ± SEM. *, *P*<0.05; **, *P*<0.01, when compared with cells treated with OS and the control siRNA.

We assessed the mRNA expression of osteoblast marker genes using qRT-PCR. As shown in Fig 3, the expression levels of *ALP*, *COL1α1*, *BSP*, *OCN*, and *OSX* were increased in cells treated with OS, and the inhibition of *BST2* by treatment with si*BST2* #1 or #2 led to decreases in the expression of these markers. Relative mRNA expression levels of *ALP*, normalized to those in cells treated with OS and the control siRNA, were 3.8 and 3.1 times lower, respectively, in cells treated with OS + si*BST2* #1 and OS + si*BST2* #2 than cells treated withOS and the control siRNA (*P*<0.01). Furthermore, the mRNA levels of *COL1α1* were 2.2 and 1.7 times lower, those of *BSP* were 1.6 and 1.3 times lower, those of *OCN* were 1.9 and 1.6 times lower, and those *OSX* were 1.5 and 1.43 times lower respectively, in cells treated with OS + si*BST2* #1 and OS + si*BST2* #2 than cells treated with OS and the siRNA control (*P*< 0.05 for all comparisons).

### *BST2* knockdown inhibited the expression of *RUNX2*

**Fig 4:**
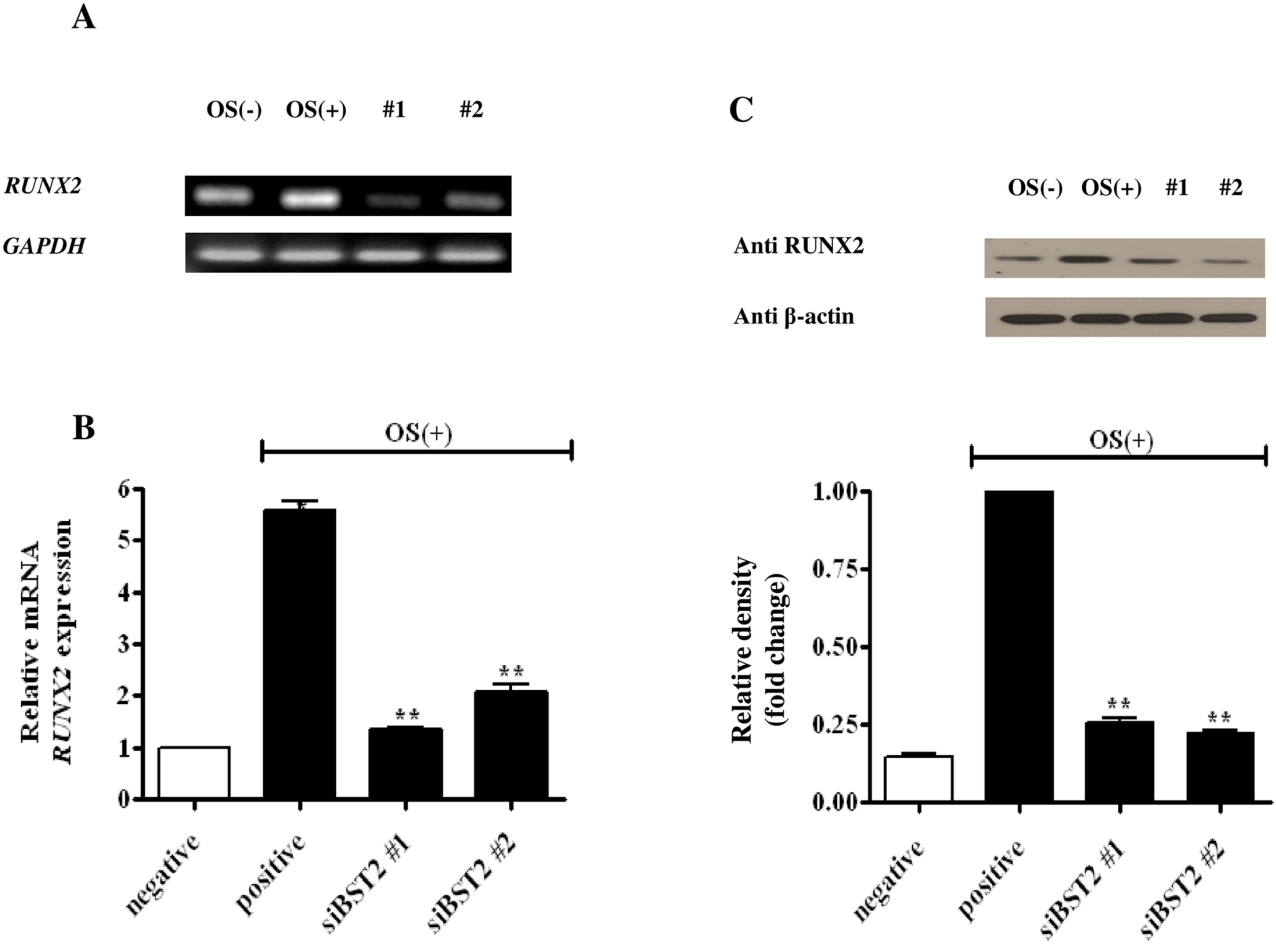
Effects of *BST2* on *RUNX2* expression. hAD-BMSCs transfected with siRNA were cultured for 5 days in the presence or absence of OS, and RT-PCR (A), qRT-PCR (B), and immunoblot analyses were performed. *GAPDH* was used as an internal standard, and the relative expression of *RUNX2* was normalized to the expression levels in of cells treated with OS alone. qRT-PCR data showed that *RUNX2* expression was reduced following *BST2* knockdown with si*BST2* #1 and #2. To determine protein expression, the cells were lysed and the proteins were analyzed by SDS-PAGE and immunoblotting (C). The band densities for RUNX2 were normalized to those for β-actin. *RUNX2* mRNA and protein expression were significantly reduced following *BST2* knockdown by siRNA. Data are expressed as means ± SEM (n = 4, **P<0.01vs. OS-treated control).

To further investigate the mechanism of *BST2*-mediated osteoblast differentiation, the expression of *RUNX2* was measured in *BST2* knockdown cells. *RUNX2* expression was higher in cells treated with OS alone, than in the untreated control; however, it was decreased in cells treated with OS and si*BST2* #1 or #2, compared with that of cells treated with OS alone (Fig 4A). As shown in Fig 4B, the relative expression of *RUNX2* mRNA (normalized to the expression level of cells treated with OS alone) was higher in cells treated with OS and the control siRNA than in untreated cells. However, relative *RUNX2* mRNA expression was significantly lower in cells treated with OS + si*BST2* #1 (4.25 ± 0.499) and OS+ si*BST2* #2 (3.53 ± 0.469) by approximately 3.9 and 2.7 times, respectively, than in cells treated with OS alone (*P*<0.01; Fig 4B). An immunoblot analysis revealed that RUNX2 protein was also highly expressed in cells treated with OS alone; however, RUNX2 expression was reduced in cells treated with OS + si*BST2* #1 and OS + si*BST2* #2, similar to the mRNA expression results for the these samples (Fig 4C).

### *BMP2* signaling promotes osteoblast differentiation via *BST2*

**Fig 5:**
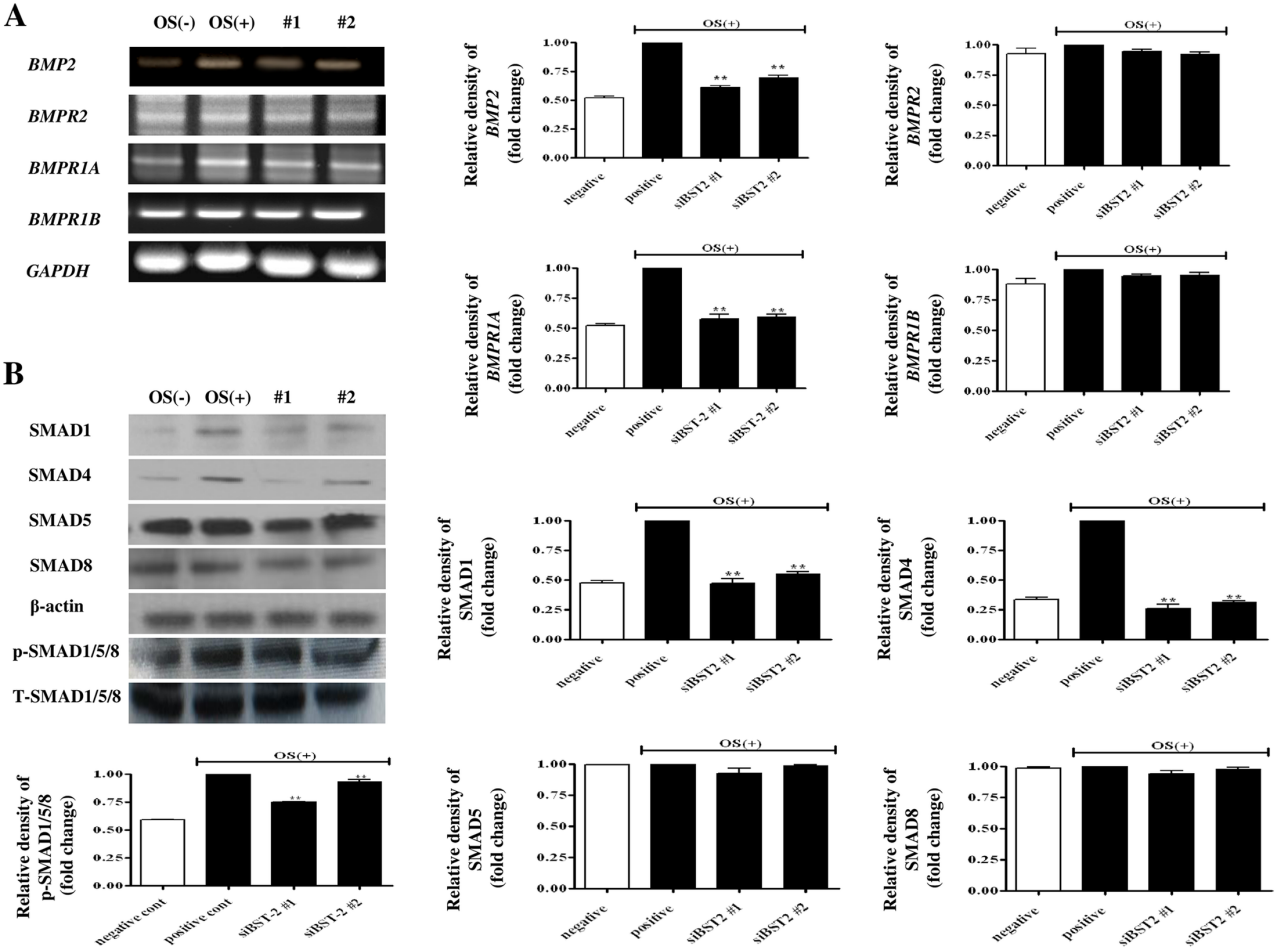
*BMP2* signaling pathway was influenced by *BST2*. hAD-BMSCs transfected with siRNA were cultured for 5 days in the presence or absence of OS, and an RT-PCR analysis was performed. *GAPDH* was used as an internal standard, and the relative expression levelsof *BMP2*, *BMPR1A*, *BMPR1B*, and *BMPR2* were normalized to the expression levels of control cells treated with OS alone. The expression levels of *BMP2* and *BMPR1A* were significantly reduced following *BST2* knockdown by siRNA (A). The cells were lysed and the proteins were analyzed by SDS-PAGE and immunoblot analyses. The band densities for SMADs 1, 4, and p-SMAD1/5/8 were normalized to those of β-actin and T-SMAD1/5/8. SMAD1, SMAD4 and p-SMAD1/5/8 expression levels were significantly reduced following *BST2* knockdown by siRNA (B). Data are expressed as means ± SEM (n = 4, **P < 0.01 vs. OS-treated controls).

To clarify whether *BST2* regulates osteoblast differentiation by modulating the *BMP2* signaling pathway, we determined the expression levels of *BMP2*, *BMPR*s, and the downstream signaling molecules *SMAD*s in *BST2* knockdown cells. *BMP2* mRNA and protein were highly expressed in cells treated with OS alone, whereas they were minimally expressed in cells treated with OS and si*BST2* #1 or #2 (Fig 5A). As shown in Fig 5A, the relative expression of *BMP2* (normalized to the expression level of cells treated with alone) was higher in cells treated with OS alone, than in untreated control cells. However, the relative levels of *BMP2* mRNA expression were significantly lower in cells treated with OS + si*BST2* #1 and OS + si*BST2* #2 by approximately 1.6 and 1.4 times, respectively, than in cells treated with OS and the control siRNA (*P*<0.01; Fig 5A). The *BMP2* receptors, *BMPR1A* showed similar results to those of *BMP2*. To further explore the mechanism by which the *BMP2* signaling pathway is involved in the *BST2-*mediated differentiation of osteoblasts, we analyzed the protein expression levels of SMADs 1 and 4 and p-SMAD1/5/8 (Fig 5B). We found significant decreases in SMAD1 protein expression levels (approximately 2.0 and 1.7 times lower, respectively) in cells treated with OS + si*BST2* #1 and OS + si*BST2* #2 compared with cells treated with OS alone (*P* < 0.01) (Fig 5B). We also found significant decreases in SMAD4 protein expression levels (approximately 4.1 and 3.4 times lower, respectively) in cells treated with OS + si*BST2* #1 and OS + si*BST2* #2 compared with cells treated with OS alone (*P* < 0.01) (Fig 5B). Additionally, the levels of p-SMAD1/5/8 expression were significantly lower in cells treated with OS + si*BST2* #1 and OS + si*BST2* #2 (approximately 1.3 and 1.1 times lower, respectively) than in cells treated with OS alone (*P* < 0.01; Fig 5B).

## Discussion

The results of this study revealed that *BST2* mediates the differentiation of hAD-BMSCs into osteoblasts by modulating osteoblast markers and the BMP2 signaling pathway. *BST2* is an antiviral gene that is overexpressed in many cancers, including breast cancer. It is critical for the invasiveness of breast cancer cells and the occurrence of metastasis *in vivo* [4, 11]. Studies of *BST2-*deficient mice imply a complex role in the regulation of viral infection [18]; it also functions as an endogenous ligand for LILRA4, also known as ILT7, and inhibits cytokine production [19], although its precise biological roles remains contentious [20]. Although the regulation of *BST2* in immune cells is becoming clear [21], a biological role for *BST2* in the process of osteoblasts differentiation from human hAD-BMSCs has not been reported. Therefore, in the present study, we investigated *BST2* expression and the effects of siRNA-mediated BST2 knockdown on differentiation makers in order to understand the mechanisms underlying osteoblast differentiation of hAD-BMSCs.

Bone formation is a typical differentiation procedure involving many osteoblast markers. Collagen and ALP are mainly involved in the early stage of this process, and OCN and BSP are mainly involved in middle and later stages. The transcription factors OSX and Runx2 are essential for osteoblast differentiation and bone formation. We found that BST2 expression was increased during osteoblast differentiation and most osteoblast markers, including OSX, exhibited decreased expression when *BST2* was knocked down by siRNA. We inferred that *BST2* has an important role in osteoblast differentiation, though the precise mechanisms were unclear. Therefore, we also examined the relationship with the key transcription factor RUNX2 and the BMP2 signaling pathway during osteoblast differentiation.

*RUNX2* interacts with various co-regulatory transcription factors, forming complexes that regulate the transcription of many bone-related factors in osteoblasts, including *ALP*, *COL1α1*, *BSP*, *OCN*, and *OSX* [10]. Furthermore, *RUNX2* may be involved in oncogenesis and the DNA damage response [22, 23]. In addition, RUNX2 function in osteoblast differentiation is affected by various regulatory genes with broad functions [10, 24]. Our results indicated that BST2 expression decreases *RUNX2* and mediates osteoblast differentiation, resulting in decreases in *ALP*, *COL1α1*, *BSP*, *OCN*, and *OSX*.

We also examined the regulatory mechanism that mediates the interaction between BST2 and the BMP2 signaling pathway. BMP2 signaling requires the oligomerization of two homodimers formed by type 1 and type 2 BMPR chains. BMP2 and BMP4 have 2 possible type 1 receptors, *BMPR1A* and *BMPR1B*, which can both oligomerize the type 2 receptor BMPR2 [25, 26]. The type 1 receptors activate BMPR-regulated SMADs (R-SMADs; SMAD1, 5, and 8) by phosphorylation. SMAD4 is known as a common partner SMAD (Co-SMAD); it passes through the nuclear membrane and forms a complex with R-SMADs [26,27]. The SMAD complex moves into the nucleus and acts as a transcription factor.

Previous studies have identified genes that act as negative regulators of the relationship between BMP-SMAD signaling during the differentiation of osteoblasts. Ishibashi et al. have reported that endoglin knockdown represses the BMP-2-induced osteoblast differentiation of periodontal ligament (PDL) cells [28]. Interestingly, there was no change in p-SMAD1/5/8 expression in endoglin-knockdown PDL cells induced by BMP2. However, they observed a change in SMAD2 expression. It is conceivable that endoglin controls the expression of BMP2-induced genes in PDL cells downstream of p-SMAD1/5/8. The authors concluded that PDL cells respond to BMP2 *via* an unusual signaling pathway that is dependent on endoglin, which is involved in osteoblast differentiation and calcification. In addition, Tan et al. reported that Smad4 knockout mice exhibit lower bone mass at up to 6 months of age. They reported negative effects of Smad4 knockout on osteoblast proliferation and function (based on mineral density, bone volume, bone formation rate, and osteoblast number) [29].

In our study, the relative mRNA expression level of *BMP2* increased in cells treated with OS, but decreased in BST2 knockdown cells. p-SMAD 1/5/8 expression was controlled by BST2 according to our results. Interestingly, SMAD1 and 4 in osteoblasts were inhibited when BST2 was knocked down. However, SMAD5 and 8 were not correlated with BST2 expression. SMAD 1, 5, and 8 are structurally highly similar, including conserved ERK/MAPK agreement phosphorylation sites in the linker region. The linker regions of SMAD1 and SMAD5 are encoded by a single exon, but the SMAD8 linker is encoded by two exons [30, 31]. Ebisawa et al. reported that there was no change in SMAD8 expression, despite SMAD1 and 5 phosphorylation, during the induction of mouse undifferentiated mesenchymal cells (C2C12) via BMP6 [32]. Sebastian et al. reported that SMAD8 is only expressed in the initial visceral endoderm, while SMAD1 and 5 were strongly expressed in mouse embryos [33]. Our results suggest that the lack of changes in SMAD5 and 8 can be explained by differences in the stages of osteoblast differentiation. Considerable evidence suggests that SMAD1, SMAD5, and SMAD8 function individually according to cell type.

In summary, our data demonstrated that *BST2* is highly expressed in osteoblasts and that *BST2* knockdown suppresses the differentiation of hAD-BMSCs into osteoblasts. Furthermore, *BST2* knockdown down-regulates the differentiation of osteoblasts *via* the BMP2 signaling pathway. This is the first demonstration that *BST2* is involved in the osteogenic differentiation of BMSCs *via* the regulation of the *BMP2* signaling pathway.
